# Deep Learning Based Instance Segmentation of Titanium Dioxide Particles in the Form of Agglomerates in Scanning Electron Microscopy

**DOI:** 10.3390/nano11040968

**Published:** 2021-04-09

**Authors:** Paul Monchot, Loïc Coquelin, Khaled Guerroudj, Nicolas Feltin, Alexandra Delvallée, Loïc Crouzier, Nicolas Fischer

**Affiliations:** 1 Data Science and Uncertainty Department, National Laboratory of Metrology and Testing, 29 Avenue Roger Hennequin, 78197 Trappes, France; khaled.creuch@gmail.com (K.G.); nicolas.fischer@lne.fr (N.F.); 2 Department of Materials Science, National Laboratory of Metrology and Testing, 29 Avenue Roger Hennequin, 78197 Trappes, France; nicolas.feltin@lne.fr (N.F.); Alexandra.Delvallee@lne.fr (A.D.); loic.crouzier@lne.fr (L.C.)

**Keywords:** scanning electron microscopy, mask R-CNN, deep learning, particle size distribution, instance segmentation, TiO_2_, agglomerate

## Abstract

The size characterization of particles present in the form of agglomerates in images measured by scanning electron microscopy (SEM) requires a powerful image segmentation tool in order to properly define the boundaries of each particle. In this work, we propose to use an algorithm from the deep statistical learning community, the Mask-RCNN, coupled with transfer learning to overcome the problem of generalization of the commonly used image processing methods such as watershed or active contour. Indeed, the adjustment of the parameters of these algorithms is almost systematically necessary and slows down the automation of the processing chain. The Mask-RCNN is adapted here to the case study and we present results obtained on titanium dioxide samples (non-spherical particles) with a level of performance evaluated by different metrics such as the DICE coefficient, which reaches an average value of 0.95 on the test images.

## 1. Introduction

**Figure 1:**
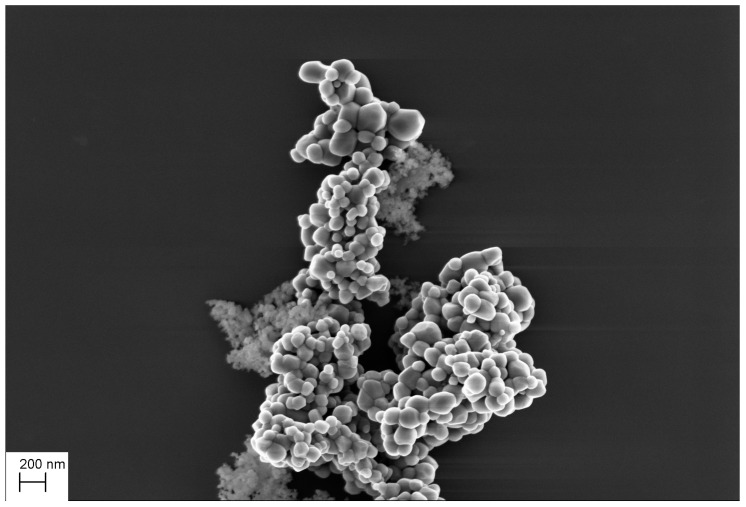
Example of the SEM image of a mixture of TiO${}_{2}$ and SiO${}_{2}$ particles.

In many cases, the estimation of particle size distribution of a nanoparticle population remains a major challenge for the industrial development of nanomaterials. Today, scanning electron microscopy (SEM) is widely used in laboratories and manufacturing industries and is considered in metrology as a reference technique capable of reliably determining the size, size distribution and shape of nanoparticles. It is a so-called direct technique because it is based on direct observations and the measurement result is directly traceable to the SI unit of length, the meter [1]. The basic principle of image analysis is: (i) identifying the contours of each nanoparticle using automatic or manual tools and (ii) determining the value of different measurands (equivalent surface diameter, Feret diameter, etc.) from surface or profile measurements. However, the reliability of the measurement is mainly related to the performance of the segmentation algorithm used to identify the nanoparticle edges. However, the determination of this contour is complicated by the natural phenomenon of agglomeration, which tends to form 3D particles bunches (Figure 1). This paper proposes a methodology to automate the size characterization of titanium dioxide particles imaged by SEM. Titanium dioxide (TiO${}_{2}$) in nanoparticulate form is produced in very large quantities for intensive use in many applications (food, paint, construction products, etc.). However, the study of the dimensional properties of titanium dioxide particles remains challenging because of their non-spherical shape and their ability for agglomeration. Due to this complexity, the characterization of this type of content is not robust and is frequently performed manually by experts in nanometrology. This task is excessively long and tedious, hence the interest in automating, even partially, the current processing chain is growing. The methodology presented in this paper relies on deep learning algorithms. Indeed, the literature in the field of deep learning has been flourishing for a few years, offering new perspectives for improvement in many areas. In particular, these recent tools have proven their effectiveness in many computer vision tasks, for which they have, in many cases, surpassed and, when appropriate, at least equaled the performance of state-of-the-art algorithms. It should be noted, however, that the major limitation to the dissemination of these algorithms has been for a long time the size of the database that had to be created in order to drive these deep networks. However, this limitation has been reduced with the development of transfer learning, which allows for use pre-trained networks, and then to train them specifically on our task with a reduced database. Starting from images acquired by scanning electron microscopy, the processing chain leading to the particle size characterization of titanium dioxide, i.e., the calculation of the equivalent diameter of each particle in the image, involves four major tasks. The first is the instance segmentation (individual particle region), followed by the steps of classifying the state of agglomeration of each particle, then, completing the partially visible particles [2], and finally computing the equivalent diameter of each particle. Even if each step must be the subject of a particular attention, the main task remains obviously the segmentation because the other processing steps depend directly on these initial performances. The learning based segmentation algorithm and its adaptation to the case study are detailed in the Section 2 and Section 3. Section 4 focuses on the analysis of the performances of the algorithm compared to human achieved segmentation.

## 2. Deep Learning Based Instance Segmentation

Image segmentation is a long time challenge for the computer vision community and many different approaches were developed. These problematics are essential in the field of nanometrology where the studies of particles are based on microscopic imaging such as Scanning Electron Microscopy imaging (SEM) or Transmission Electron Microscopy imaging (TEM). We will focus in this section on the various strategies of automation of image segmentation.

A first approach of image segmentation is based on classic image processing techniques, among which we can find watershed methods and its variants and adaptations [3,4,5], histogram based methods [6], graph-cut methods [7], or active contour methods [8]. These techniques present the advantage to be efficient but need a strong parameter tuning in order to make them work properly on specific images. Indeed, before applying any of these algorithms, it is necessary to apply numerous image processing techniques such as filtering, thresholding, morphological operations,… All of these techniques require specific tuning to achieve high level instance segmentation accuracy on a given image. Thus, the major drawback of these methods is their lack of genericity.

A second approach is based on software tools such as ImageJ [9], Ilastik [10] or self developed software such as SEMseg [5]. These types of software propose a wide range of functionalities allowing the user to process the image without implementing code or having a deeper knowledge of image processing algorithms and techniques, but does not offer a sufficient level of automation of the specific task to the end-users.

A third approach is based on the development of image processing workflows, mixing general image processing methods and machine learning methods such as the EM algorithm [11] or the K-means algorithm [12]. These types of workflows show strong performances at the cost of parameter tuning (lack of genericity) or include strong prior hypothesis, such as shapes to be detected for example.

The final category of methods is based on deep-learning algorithms which we can separate on two distinct sub-categories: semantic segmentation and instance segmentation methods. Semantic segmentation methods are mainly using auto-encoders network such as UNet [13]. These algorithms are powerful, generic and highly accurate. These methods propose to recreate the input image as a segmentation map where each pixel represents a class label of the detected object. While semantic methods are adapted to numerous cases of study such as medical images [14,15], mineral characterization [16] or meteorology [17], they can not be applied to our images, where TiO${}_{2}$ particles tend to agglomerate.

Therefore, instance segmentation methods seem to be adapted to our case study and the specificity of the acquired TiO${}_{2}$ agglomerate images. These methods offer genericity, a separate detection of every instance and robustness, but need a large amount of training data, which can be difficult to obtain. Indeed, some of the TiO${}_{2}$ particle SEM images can show more than 500 different instances which need to be manually segmented.

The main algorithm used for instance segmentation is an algorithm developed by the Facebook Research Team (FAIR) called “Mask-RCNN” [18]. It has been tested in various fields such as medical image analysis (brain tumor detection [19], nucleus detection in microscopy [20], detection of lung nodule [21]), satellite images analysis [22], very high spatial resolution aerial imagery [23] or astronomy [24] to name but a few examples. This algorithm showed also promising results on STM images of nanoparticles [25].

The Mask-RCNN has become a standard in the deep learning community, being both generic and efficient. It can be seen as a two-stage algorithm: the first stage generates candidate object bounding boxes and the second stage predicts the class, bounding box, and binary mask for each region of interest (ROI). The convolutional backbone performs feature extraction over the whole input image and the head part ensuring both bounding-box recognition (classification and regression) and the prediction of the binary masks. A specific network called the Region Proposal Network (RPN), as its name indicates, computes the region proposals by using a sliding window (called anchors) technique directly on the feature maps (backbone outputs) to generate the bounding boxes. The proposed bounding boxes, having different sizes, are mapped to fixed spatial resolution using standard bi-linear interpolation to produce processed feature maps. After this, a more common pipeline is setup so that, for each ROI, fully connected layers (FC) serve to predict the class and to refine the associated bounding box, and, simultaneously, a fully convolutional network (FCN) produces binary masks. Residual learning network (ResNet) [26] is the standard backbone and it is coupled with a Feature Pyramid Network (FPN) [27] for improving the representation of the objects at multiple scales and in the meantime improving particularly the accuracy.

For more insights on the algorithm, please refer to the original article [18].

## 3. Adaptation of the Mask RCNN for the Detection of TiO${}_{2}$ Particles Measured by SEM

The key step in any development using statistical learning is the creation of a function-specific database from which the network will learn how to make predictions on new data, never processed by the network, hence the systematic breakdown of entries into a learning, testing and validation database. If the Mask-RCNN algorithm is very powerful for computer vision tasks, it is nonetheless resource-intensive, in other words, its training requires a very large image database (several million samples). In our case, it is simply unthinkable to manually segment so many images. Section 3.1 describes the function-specific database created for the segmentation task and Section 3.2 and Section 3.4 respectively present two approaches to use the Mask-RCNN with this reduced database, namely data augmentation and transfer learning. Section 3.3 focuses on hyper-parameters and network architecture adjustment to achieve high level accuracy in the predictions of the binary masks. Finally, Section 3.5 details the training strategy and the software and hardware specifications used during the training phase.

### 3.1. Creating the Function-Specific Database

**Figure 2:**
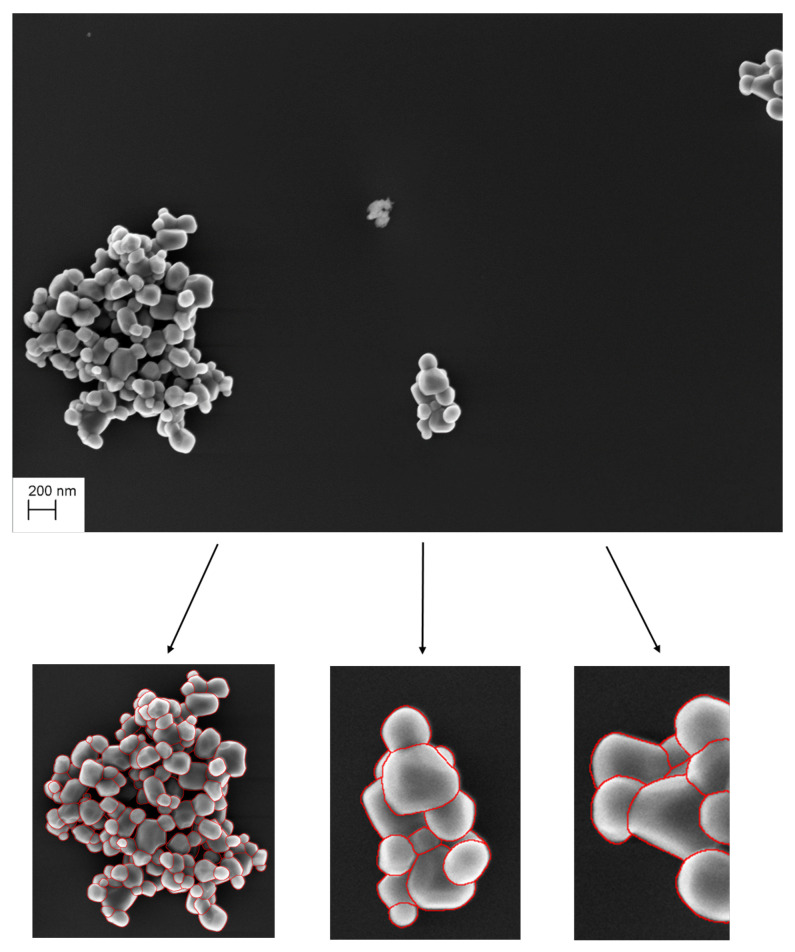
Measured SEM image (**up figure**) and its annotated agglomerates (**down figures**).

The database built to perform the segmentation is currently composed of 77 images manually segmented by four operators trained by nanometrology experts representing 5947 particles of TiO${}_{2}$. These same experts then validated the masks thus produced before their incorporation into the database. The input of the algorithm is a tensor of size W×L×C×N: *W* and *L* correspond to the width and length in number of pixels of each SEM image (*W* = 2048, *L* = 1536), *C* corresponds to the number of channels of the image (*C* = 1, SEM measurement returns a greyscale image) and finally N represents the number of available samples (*N* = 77). The annotations for each image in the reference database are stored in a JSON file which allows for representing a class instance (here a particle in the image) by an identifier and a binary mask (corresponding to the reference segmentation produced by each operator). Figure 2 shows an SEM image of agglomerated titanium dioxide particles and its annotated agglomerates (manual segmentation).

### 3.2. Data Augmentation

Data augmentation is very common in statistical learning, especially when a small database is available. This step should improve the robustness of the algorithm and artificially enrich the initial database by applying mathematical transformations on the input images such as rotation, translation, cutting,… The data enhancement proposed here is application specific. The main objective is to create “fake” data images as close as possible to real images without requiring any post processing to obtain the reference segmentation. We also want to avoid introducing any underlying logic in the generated images.

**Figure 3:**
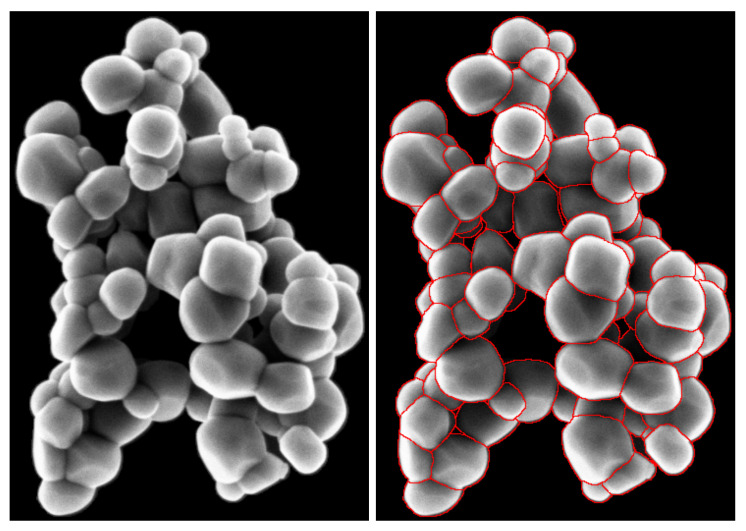
Extracted agglomerate of TiO${}_{2}$ particles (**left figure**) and its segmented version (**right figure**).

Starting from the reference segmented images, each agglomerate of each segmented images is extracted to build a library of agglomerates (Figure 3). For example, in Figure 2, three agglomerates are extracted. The data enhancement then consists of simulating new images by first randomly applying flipping and rotation to these clusters, and, second, randomly positioning these clusters of particles on different empty SEM backgrounds (with or without matrices, the matrix may be made up of other particles such as silicon dioxide particles SiO${}_{2}$).

**Figure 4:**
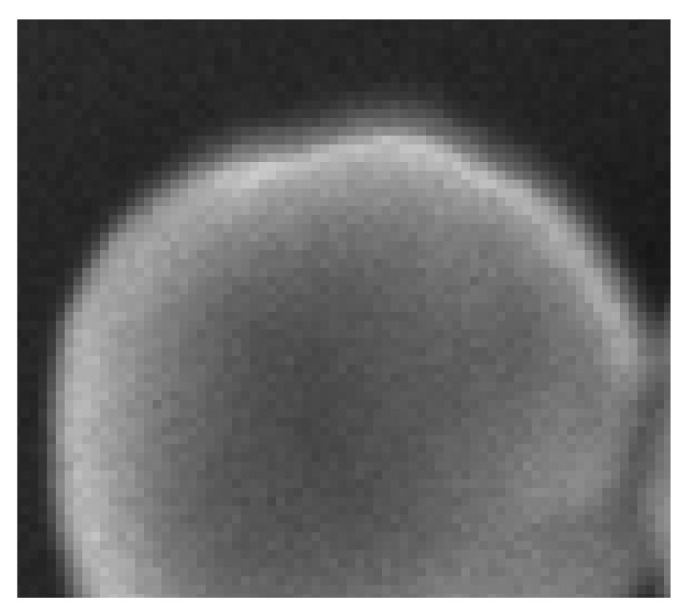
Particle border visualization with ×16 zoom.

It is important to note that, during the phase of placement, no superposition among agglomerates should occur. While superposing agglomerates can create a totally new configuration of TiO${}_{2}$ particles, it also produces unrealistic borders. Indeed, agglomerate borders are usually spread over couple pixels due to the angle of incidence of the electron beam of the SEM (Figure 4).

**Figure 5:**
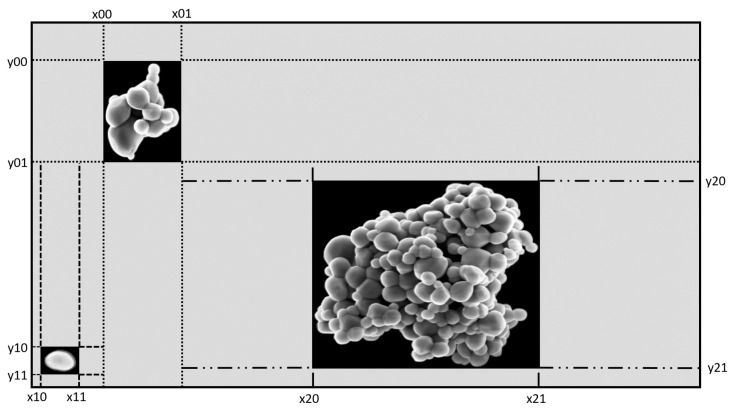
Schematic of the image simulation procedure.

Thus, after inserting the first agglomerate in the frame, eight different sub-frames are “created” where the following agglomerate will be placed randomly (Figure 5). The procedure continues until there is no more space available or when the maximum number of agglomerates to insert has been reached. Finally, a 5 × 5 median filter over all the agglomerates borders is applied in order to smooth the transition between the background frame and the inserted agglomerates.

**Figure 6:**
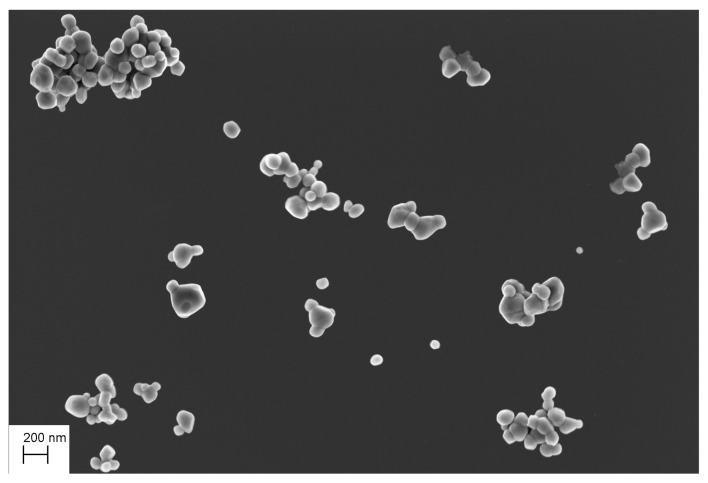
Simulated SEM image of TiO${}_{2}$ particles.

The proposed data augmentation procedure is coupled with more common data augmentation strategy such as randomly applying one or multiple transformations among horizontal flip, vertical flip, Gaussian blurring, contrast normalization, additive Gaussian noise, and pixel value multiplication. Figure 6 shows an example of a simulated image.

### 3.3. Hyper-Parameters and Network Architecture Adjustments

While the work of creating the database is tedious, the choice of network hyper-parameters remains a matter for experts. Among the most common hyper-parameters in computer vision, one can list the learning rate, the optimization solver (Stochastic Gradient Descent (SGD), ADAM [28], Momentum,…), the intrinsic parameters of each solver (regularization parameter, number of epochs,…), the stopping criterion (when should I stop learning),…. For these rather generic parameters, it is advisable to rely on experts in the field and to read the good practice guides, review papers such as [29,30] and numerical comparisons [31,32] in order to decide on a set of relevant hyper-parameters. However, the task of titanium dioxide particle segmentation is very different from the tasks for which such algorithms are used. It will therefore be necessary to adapt this one accordingly. The SEM images under study involve a large number of particles in each image, sometimes several hundred or even thousands. However, the algorithm was initially developed to detect people, cars, etc… in other words, the number of class instances per image is drastically different from that of the original task.

One of the first adaptations of the algorithm was therefore made to the Region Proposal Network (RPN). The objective of the RPN is to scan (by means of anchors) over all the feature maps extracted by the backbone network and scores the presence of object and estimates refining deltas for a better fitting of the anchor to the detected object.

**Figure 7:**
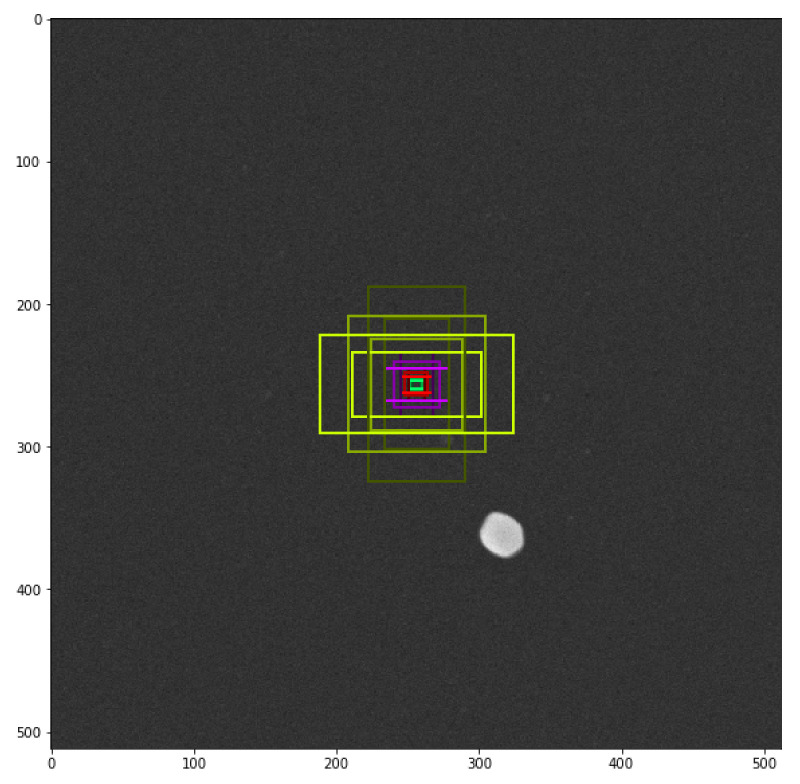
Different anchors sizes and shape at one location.

Anchors allow the network to detect multiple object of different sizes and scales. Figure 7 displays at one location the set of anchors specified by the hyper-parameters specifying the anchor sizes, the anchor aspect ratio.

It is then necessary to adapt the sizes and aspect ratios of anchors to the objects we want to detect. We can also specify the number of trained anchors which must be of the same magnitude as the number of objects to detect and the anchor stride which specifies the number of pixel between two anchor positions.

Thus, we adapt these hyper-parameters to the case study:

- The sizes of anchors were modified according to the minimum and maximum size of TiO${}_{2}$ particles: [8, 16, 32, 64, 96] (in pixels);
- The number of trained anchors was modified according to the maximum number of particle in one image: 1024;
- The stride between two consecutive anchors was set to 1 due to the agglomeration phenomenon.

The second part of adjustments was driven by the wish to improve the segmentation accuracy produced by the network. Indeed, this is the most critical aspect for our specific task. In order to do so, we modified the FPN Mask Graph, the network responsible to produce the final predicted mask. We simply augmented the “resolution” of the predicted mask by adding an additional transposed convolutional layer. This modification allowed to improve slightly the segmentation performances (improvement of 0.5 over the dice coefficient).

Finally, we adjust several hyper-parameters of the algorithm to fit the specificities of our segmentation task, such as the number of maximum ground truth instance (corresponding to the maximum instance to detect in one image), the number of trained ROIs (corresponding to the number of ROIs to generate and to feed the head networks), and the use of mini-mask (SEM images have high resolution, it is therefore mandatory to work with mini-mask), the size of mini-mask roughly corresponding to the maximum size of a single particle (in our case, 96 pixels) and, finally, the mean pixel value for image normalization (calculated over all the training dataset). Other hyper-parameters linked with training strategy are detailed in Section 3.5.

### 3.4. Transfer Learning

Transfer learning is a deep learning technique allowing one to avoid training its network from scratch. Indeed, transfer learning consists of initializing the network weights from weights of another network (having the same architecture) but trained for another task. Then, it is only needed to fine-tune these weights according to the specific task. This technique is possible due to the fact that, during training, neural networks “learn” how to extract low-level features on shallow layers, while task specific features are extracted thanks to more deeper layers.

The most common approach in transfer learning is to start the training by focusing only on the network heads (transferred weights in the firsts layers are kept fixed at this stage), and, then, after a chosen number of epochs, to train the whole network for the specific task (the whole weights are updated), in this case the detection of titanium dioxide particles. The weights transferred are from the algorithm trained on the MS COCO database [33]. This database contains 91 distinct object categories and nearly 2,500,000 instances annotated in 328,000 images. At this stage, it is difficult to decide how many samples are required in the learning process to achieve a given level of performance. Simply put, the size of the database is increased until a satisfactory level of performance is achieved. In our case, satisfactory means a segmentation close to what the human operator would do. Indeed, if the automatic segmentation is almost equivalent to what the human operator would do, a simple and very short correction pass will allow us to obtain an accurate segmentation of the particle in a reduced time.

Our strategy of transfer learning in our application follows the common approach previously explained. Details about the training strategy are available in Section 3.5.

### 3.5. Network Training

This section details the training strategy of the Mask R-CNN and the software and hardware specifications used during the training process.

Our train set was constituted of 699 images, from which 77 were “real” images and 622 were images produced via the data augmentation strategy.

As explained previously, the network heads were trained during 38 epochs with a learning rate of 0.001, four epochs with a learning rate of 0.0001. Then, we trained the all network during 28 epochs with a learning rate of 0.001 and finally seven epochs with a learning rate of 0.0001.

Each epoch is made up of 698 steps, where each step uses two images per GPU during the head training phase and one image per GPU during the entire network training phase.

We used a stochastic gradient descent optimizer with a momentum of 0.9 and with a gradient norm clipping of 5.0. We used L${}_{2}$ regularization with a weight decay [34] of 0.0001.

For more details about data augmentation strategy and transfer learning, please refer to the respective section.

Our network was trained on a GPU NVIDIA GeForce RTX 2080 with 8 GB of memory (with the 26.21.14.4575 version of the driver).

We used the 2.1.6 version of Keras with the 1.8.0 version of Tensorflow GPU library. We used the 7.6.5 version of Cudnn and the 9.0 version of Cudatoolkit.

## 4. Results

### 4.1. Test Set

**Figure 8:**
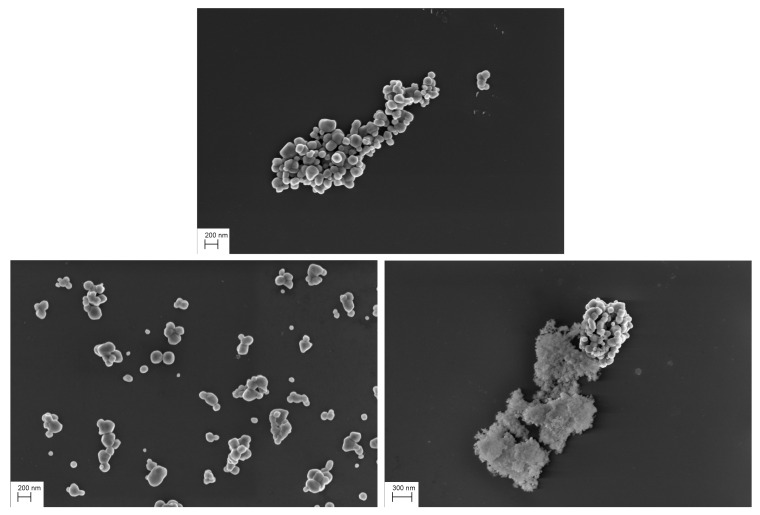
Test SEM images, agglomerate TiO${}_{2}$ particles (**top figure**), scattered TiO${}_{2}$ particles (**bottom left figure**) and with a mixture of SiO${}_{2}$ and TiO${}_{2}$ particles (**bottom right figure**).

The test set is made of 19 images representing 3741 particles of TiO${}_{2}$. These 19 images are representing the different types of configuration we can encounter in our field of study. TiO${}_{2}$ particles can be present in a form of aggregate, scattered or in the presence of matrix of other types of particles (for example, with a matrix of SiO${}_{2}$ particles). Figure 8 displays three test images representing the different type of particle configurations.

**Figure 9:**
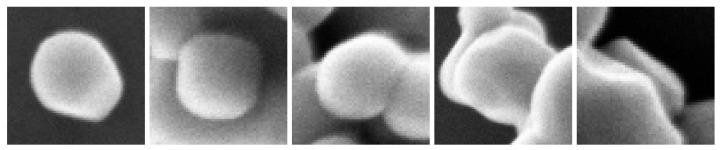
TiO${}_{2}$ particles in different configuration: isolated, complete, touch complete, masked and unusable (from **left** to **right**).

The different images also show a great diversity of particle layout (Figure 9):

- Isolated: the particle is completely imaged and located outside of an agglomerate,
- Complete: the particle is completely imaged and located in or near an agglomerate,
- Touch complete: the particle is completely imaged but interlocked with another particle (between the complete state and the masked state),
- Masked: the particle is partly hidden by an other particle,
- Unusable: the particle is masked by an other particle with a very small visible area (less than 40 percent of its area is imaged and, therefore, does not constitute an interest for our purpose of size distribution measurement)

The following results will be displayed accordingly to these configurations.

### 4.2. Result Analysis

In order to evaluate the detection performance of the network, the mAP metric from the COCOApi was used. Then, the Sørensen–Dice coefficient [35] between the detected particle and the corresponding reference segmented particle is used in order to evaluate the segmentation performance of the network. Finally, a focus will be made on the impact of these metrics on the final measurands of the particles such as the projected diameter, the perimeter, the area, or the Feret diameter.

As specified previously, our test set consists of 3741 particles of TiO${}_{2}$, showing a great diversity of configurations (isolated particles, complete particles, masked particles,…), shapes and sizes. The equivalent projected diameter of our reference particles is between 2 and 128 pixels (some particles are almost entirely hidden by other particles inside agglomerates, they are classified as “unusable”).

Table 1 explicits the different parameters used during the test phase.

#### 4.2.1. Performances

The test evaluation was performed using the same hardware as for the training:

- GPU NVIDIA GeForce RTX 2080 with 8 GB of memory
- CPU Intel Core i9 3.60 GHz
- RAM 32Go

The segmentation task over the entire test set (19 images) was performed in 110 s, equivalent to 5.79 s per image or 0.035 s per detected particle. To give some points of comparison, a segmentation performed manually takes about 15 to 30 s per particle. If extended to the entire test set, it takes at least 15 × 3741 = 56,115 s (~15.6 h).

#### 4.2.2. Visual Analysis

**Figure 10:**
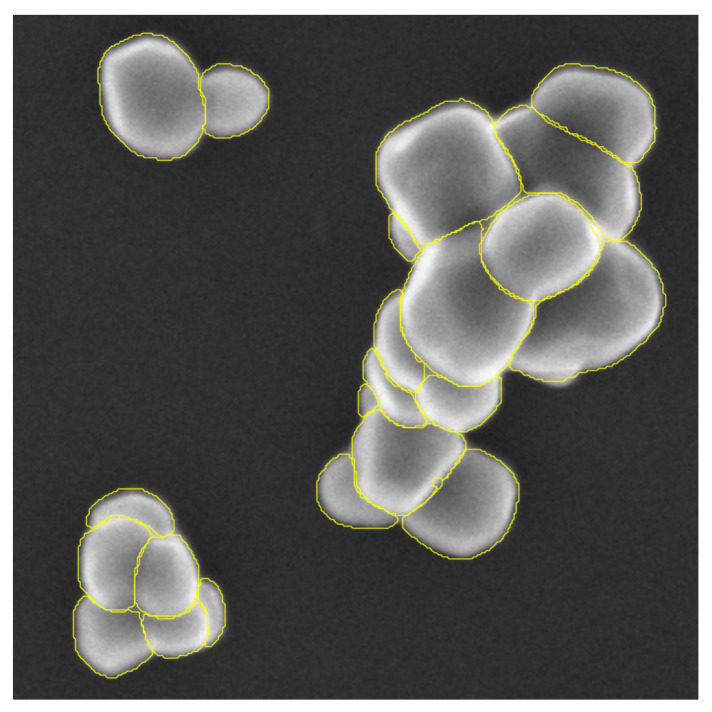
Predicted segmentation over SEM image of scattered TiO${}_{2}$ particles, zoom ×8.

**Figure 11:**
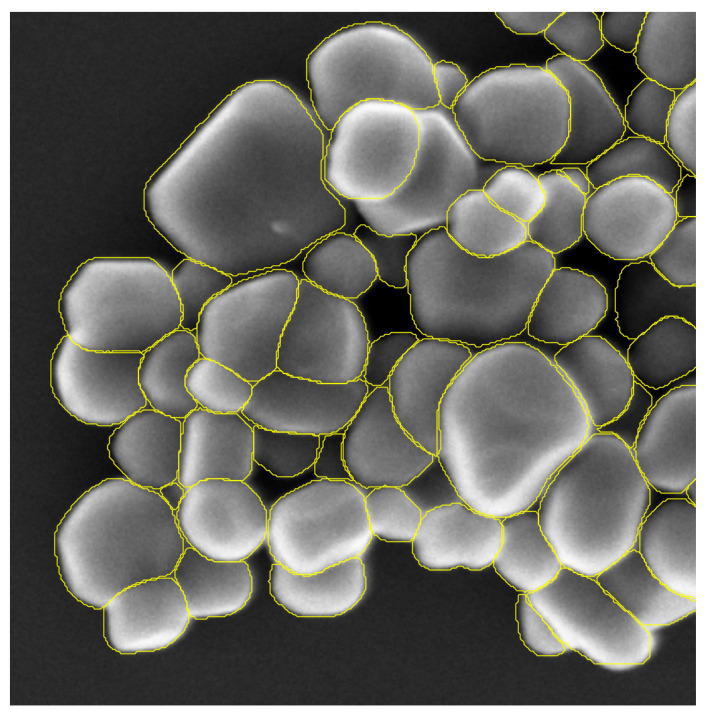
Predicted segmentation over SEM image of agglomerated TiO${}_{2}$ particles, zoom ×8.

**Figure 12:**
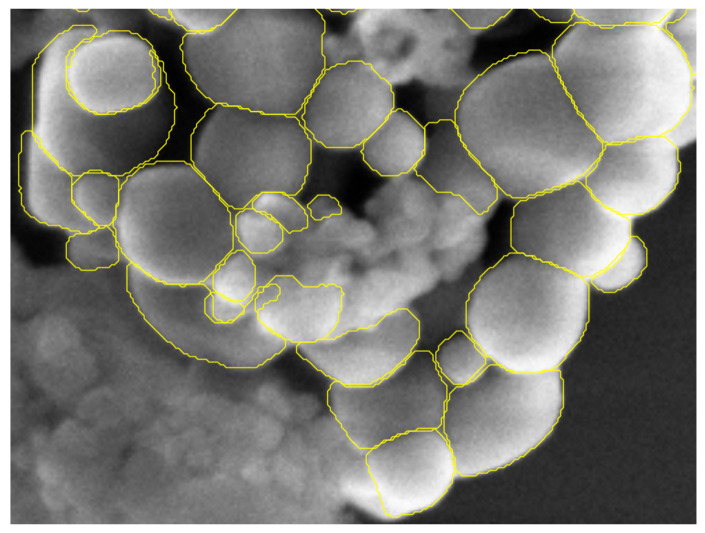
Predicted segmentation over SEM image of TiO${}_{2}$ particles in the presence of SiO${}_{2}$ particles, zoom ×8.

Figure 10, Figure 11 and Figure 12 provide a first visual hint of the segmentation performance over three images (images with a ×8 zoom for better visualization).

Visually, we can note several interesting points. First, almost all particles were detected over the images, except for one particle in Figure 11 located at the top of the image. Secondly, we can see that only three false positives were inferred: two in Figure 12 located in the SiO${}_{2}$ matrix and one in Figure 11 in the darker area on the right side of the image. As previously noted, Figure 12 shows a mixture of SiO${}_{2}$ and TiO${}_{2}$ particles. At these locations, we can note a degradation of the segmentation borders. This slight lower performance on these areas can be explained by the composition of the training database where only 23 images show a matrix of other types of particles (recalling that the training database was constituted of 699 images). We can also observe that the Mask R-CNN produced segmentation masks of high precision inside the agglomerates. Borders between different particles are clear and well defined, no overlaps between the different segmentation masks. Finally, it is more difficult to appreciate the performance of the network over segmentation mask borders on the agglomerate periphery. Indeed, as Figure 4 is showing, the borders of agglomerates are diffused over a few pixels. This is mainly due to electron sample interaction phenomena. Therefore, the segmentation performance on these areas can not be evaluated with the naked eye, and it is necessary to evaluate metrics by comparing the measured segmentation with a reference (produced by operators under expert supervision of directly by the experts).

We can also underline the fact that the segmentation mask shows saw-tooth borders. This phenomenon is due to the rescaling step applied to the inferred segmentation masks in order to retrieve the original size.

#### 4.2.3. Detection Results

Table 2 summarizes the number of detected particles in the test set versus the number of particles in the reference segmentation (created by manual segmentation and validated by experts).

Overall, the Mask R-CNN algorithm was able to detect around 84 percent of particles. This value reaches 97 percent when we only keep useful particles (i.e., not classified as “unusable”). The detection performance is piloted by the hyper-parameter controlling the detection threshold and thus can be modified. The threshold value is set in order to detect almost all useful particle instances without generating false positives (especially in images presenting a matrix).

To complete the evaluation of the detection performance of our network, we calculate the mean Average Precision [36] (mAP). mAP is a popular metric allowing one to evaluate the detection performances of an algorithm by computing the precision/recall curve over different IoU thresholds. Table 3 presents the results of this evaluation.

#### 4.2.4. Segmentation Results

Previously, we detailed the detection performance of the algorithm thanks to the detection scores and the mAP metric. Now, we want to detail the segmentation performance of the algorithm by measuring the Dice coefficient over the detected particles. These results are shown below and are expressed according to the type of the detected particle.

Table 4 details the dice coefficient statistics over detected particles according to their type (complete, touch complete, masked, or unusable).

Once again, the global performance of the network is satisfactory with a global dice coefficient of 0.936. The coefficient reaches 0.95 over useful particles. The dice coefficient drops to 0.90 when we are looking at “unusable” particles. This can be explained by looking at the definition of particles classified as “unusable”. We recall that they are masked particles, whose visible area is very small (less than 40 percent of the particle area), which makes them more difficult to segment.

**Figure 13:**
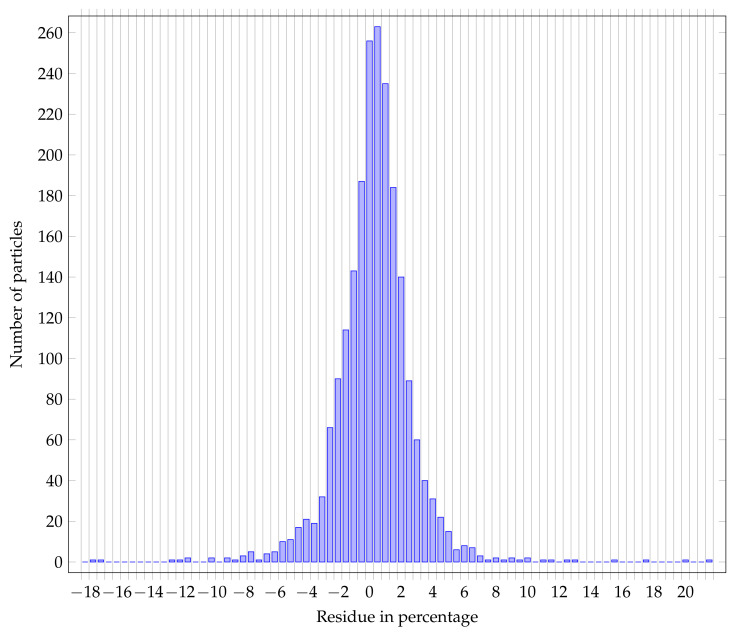
Residue distribution of equivalent surface diameter (in percentage).

**Figure 14:**
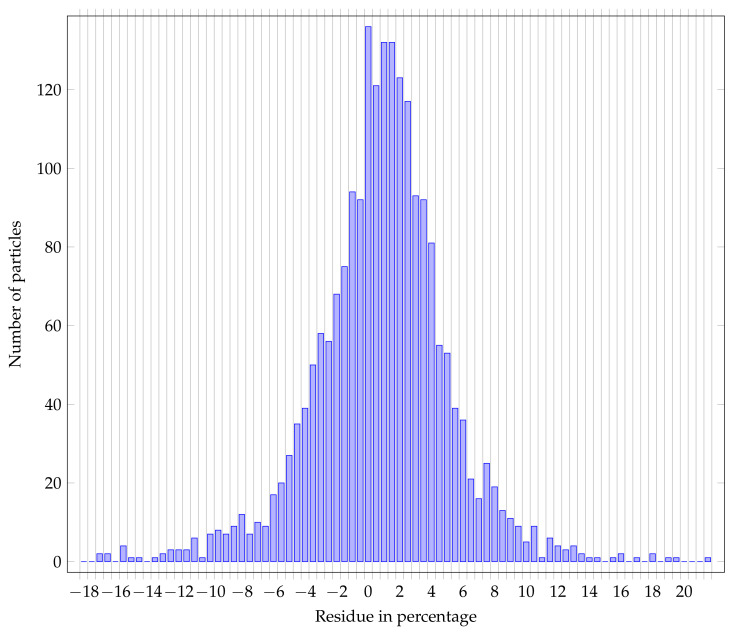
Residue distribution of particle area (in percentage).

Finally, one can wonder how the dice coefficient is translated to the final measurands which are the area, the equivalent surface diameter, the Feret min diameter [37], the Feret max diameter, and the perimeter. Histogram Figure 13 shows the residual distribution of the equivalent surface diameter of detected particles classified as useful in percentage and histogram Figure 14 shows the residual distribution for the area of the useful detected particles in percentage.

The distribution shows a mean of 0.56 and a standard deviation of 2.48. Moreover, more than 96 percent of measurements show an error of less than 5 percent and 51 percent of measurements show an error of less than 1 percent (compared to the manual reference produced by operators).

The distribution shows a mean of 1.06 and a standard deviation of 4.93.

Two observations can be made over these two distributions:

- Bias: the residual distributions are globally centered on 0, the bias could come from the network itself, or from a bias in the reference annotations.
- Variance: the variance of the residual distribution is higher over the area measurand than for the Feret min diameter; in fact, a small error over the radius has a squared influence over the calculated area,

We should highlight that the hardware is currently the main performance limitation. Stronger architectural modifications of the Mask R-CNN network will allow a sensitive performance improvement but need more GPU memory to process it.

## 5. Conclusions

Thus, through this work, we successfully showed that the instance segmentation deep learning algorithm Mask R-CNN shows robustness and high performance when applied to SEM images of TiO${}_{2}$ particles with the presence of big agglomerates. These performances were achieved by performing hyper-parameters tuning and through a small architecture modifications. We also proposed a new method of data augmentation adapted to our specific task and will take another level of relevance when applied to SEM images containing different types of particles. Finally, for SEM image processing, we highlight that deep learning algorithms challenge human performance in terms of precision but with a much higher genericity, repeatability, speed of processing and robustness.

This work aims to fully automate the particle size characterization chain in scanning electron microscopy. Coupled with the work of [2] to complete the masked particles within the agglomerates and the work undertaken on the classification of the agglomeration state of the individual particles in the SEM image (depending on the state of the particles, the processing differs, e.g., the masked particles go through the completion phase before the calculation of the equivalent diameter as shown in the diagram below), a fully automatic characterization chain is achieved easily. However, if this automatic characterization process materializes, other questions are raised at the same time, including the confidence and uncertainty associated with these learning-based methods. Indeed, the use of these tools raises questions about the control that can be established over the predictions and thus about our ability to understand how uncertainty propagates within these types of networks. It is therefore necessary to establish a methodological framework for quantifying the sensitivity, robustness, and more broadly the uncertainty associated with these types of algorithms. Work is currently under way on this topic in order to be able to produce a level of confidence associated with the estimation of the particle size distribution on SEM samples. The current comparison is therefore limited to the comparison between the expertise and the predictions of the Mask-RCNN, and the dissemination of these methods, assuming that, if the results are equivalent, their use under control can be favoured, given the time savings they produce.

Finally, in this article, we did not tackle the post processing techniques in order to improve segmentation results. Several approaches exist in order to post process the optimal border location [38]. It will be developed in a future work.

## Figures and Tables

**Table 1:** Parameters used during the testing phase.

Parameter	Value	Description
Resizing mode	Pad 64	No resizing of the image at inference time
Minimum detection confidence	0.5	Threshold over class probability above which we keep a detection
Detection NMS Threshold	0.3	Threshold for deciding whether boxes overlap too much w.r.t IOU.
Detection post NMS ROIs	1000	Maximum number of ROIs to keep after NMS
Detection maximum instances	512	Maximum number of final detection
Number of image per GPU	1	The number of image your GPU memory can fit

**Table 2:** Number of detected particles on the test set according to their type.

Detected Particles	All	Complete	Touch Complete	Masked	Unusable
Reference	3741	341	515	1302	1583
Measured	3135	339	495	1253	1048
Percentage	83.80	99.41	96.11	96.23	66.2

**Table 3:** AP score for detection evaluation of Mask R-CNN over SEM images of TiO${}_{2}$ particles.

	mAP	AP${}_{50}$	AP${}_{75}$	AP${}_{90}$
Score	60.6	84.6	71.0	21.5

**Table 4:** Sørensen–Dice coefficient statistics over detected particles.

Dice Metric	Mean	Median	Std	Min	Max
All	0.936	0.948	0.040	0.750	0.989
Complete	0.953	0.959	0.024	0.819	0.987
Touch Complete	0.953	0.958	0.022	0.858	0.986
Masked	0.949	0.957	0.026	0.765	0.989
Unusable	0.903	0.913	0.046	0.756	0.975

## Data Availability

The data presented in this study are available on request from the corresponding author. The data are not publicly available due to industrial confidentiality.

## Abbreviations

The following abbreviations are used in this manuscript:

SEM	Scanning Electron Microscopy
R-CNN	Regional Convolutional Neural Network
ROI	Region of Interest
RPN	Regional Proposal Network
FPN	Feature Pyramid Network
TiO_2_	Titanium Dioxide
TEM	Transmission Electron Microscope
STM	Scanning Tunneling Microscope
FCN	Fully Connected Network
ResNet	Residual Network
SGD	Stochastic Gradient Descent
